# Genome Organization and Adaptive Potential of Archetypal Organophosphate Degrading *Sphingobium fuliginis* ATCC 27551

**DOI:** 10.1093/gbe/evz189

**Published:** 2019-08-27

**Authors:** Sarwar Azam, Sunil Parthasarathy, Chhaya Singh, Shakti Kumar, Dayananda Siddavattam

**Affiliations:** 1 National Institute of Animal Biotechnology, Hyderabad, India; 2 Department of Animal Biology, School of Life Sciences, University of Hyderabad, India

**Keywords:** biodegradation, organophosphate hydrolase, genome sequence, plasmids, mobile elements, horizontal gene transfer

## Abstract

*Sphingobium fuliginis* ATCC 27551, previously classified as *Flavobacterium* sp. ATCC 27551, degrades neurotoxic organophosphate insecticides and nerve agents through the activity of a membrane-associated organophosphate hydrolase. This study was designed to determine the complete genome sequence of *S. fuliginis* ATCC 27551 to unravel its degradative potential and adaptability to harsh environments. The 5,414,624 bp genome with a GC content of 64.4% is distributed between two chromosomes and four plasmids and encodes 5,557 proteins. Of the four plasmids, designated as pSF1, pSF2, pSF3, and pSF4, only two (pSF1 and pSF2) are self-transmissible and contained the complete genetic repertoire for a T4SS. The other two plasmids (pSF3 and pSF4) are mobilizable and both showed the presence of an *oriT* and relaxase-encoding sequences. The sequence of plasmid pSF3 coincided with the previously determined sequence of pPDL2 and included an *opd* gene encoding organophosphate hydrolase as a part of the mobile element. About 15,455 orthologous clusters were identified from among the cumulatively annotated genes of 49 *Sphingobium* species. Phylogenetic analysis done using the core genome consisting of 802 orthologous clusters revealed a close relationship between *S. fuliginis* ATCC 27551 and bacteria capable of degradation of polyaromatic hydrocarbon compounds. Genes coding for transposases, efflux pumps conferring resistance to heavy metals, and TonR-type outer membrane receptors are selectively enriched in the genome of *S. fuliginis* ATCC 27551 and appear to contribute to the adaptive potential of the organism to challenging and harsh environments.

## Introduction

Agricultural soils contain high amounts of insecticide residues due to repeated and indiscriminate use of insecticides. Microorganisms living in soils gain access to these xenobiotic compounds, hitherto unknown to the native environment. Some of the microbial enzymes show promiscuous activity toward the accumulated residues due to the existence of structural similarities with their cognate substrates (Guzman et al. 2019). Such promiscuity contributes to the evolution of new enzymes/degradation pathways required for the degradation/mineralization of these toxic substances (Kolvenbach et al. 2014).

Organophosphate (OP) insecticides, which are used as substitutes for the most persistent organochloride insecticides, have potentially neurotoxic effects and adversely impact on human and animal health (Bouchard et al. 2011). *Sphingobium fuliginis* ATCC 27551, previously classified as *Flavobacterium* sp. ATCC 27551, degrades a wide range of OP insecticides due to the existence of a plasmid-encoded triesterase, designated as organophosphate hydrolase (OPH) (Sethunathan and Yoshida 1973; Mulbry et al. 1986; Mulbry and Karns 1989; Kawahara 2010). *Sphingobiumfuliginis* ATCC 27551 contains four indigenous plasmids, of which only plasmid pPDL2 carries the *opd* gene. The physiological significance of the other three plasmids is unknown. Although the ability of *S. fuliginis* ATCC 27551 to degrade OP pesticides is known, due to the lack of genome information its potential has not yet been fully exploited to remediate OP-polluted environments. The present study reports the complete genome sequence of *S. fuliginis* ATCC 27551 and its adaptive potential to harsh environments.

## Materials and Methods

The whole genome of *S. fuliginis* ATCC 27551 was sequenced using Pacific Biosciences (Pacbio RSII) and Illumina platforms (MiSeq). PacBio reads were assembled using Canu, HGAP2, and HGAP3 pipelines and circularized by circlator v1.4.0 (Hunt et al. 2015). Processed MiSeq data were mapped onto assemblies using bowtie2 (Langmead and Salzberg 2012) and Canu assembly was chosen for the final assembly. The RAST server (Brettin et al. 2015) was used for structural and functional annotation. COG (Cluster of Orthologous Groups) profiles were assigned to the genes using RPS-BLAST v2.4.1. Only genes with at least 25% identity, 70% alignment length, and an e-value cut-off of 0.01 were taken into consideration while assigning COG functional groups. Proteins coded by these genes and the proteome of 48 species of the *Sphingobium* genus downloaded from PATRIC database were used for orthology analysis using Orthomcl v1.4 (Li et al. 2003). The phylogenetic tree was constructed using RAxML (Stamatakis 2014). Plasmid pPDL2 (NCBI: JX312671.1) of *S. fuliginis* ATCC27551 (Pandeeti et al. 2012) was compared with the in-house assembled plasmids using the Mummer3 package (Kurtz et al. 2004). The detailed methodology is given in the Supplementary Material online.

## Results and Discussion

The 796 Mb data generated through a single SMRT cell of PacBio were filtered to select reads with a length of 500 bp and above. After filtration, 133,313 reads were obtained and of these, the largest read was of 48.2 kb. If the approximate size of the *S. fuliginis* ATCC 27551 genome is assumed to be ∼5 Mb, the total filtered data of 795.2 Mb amounts to a 159-fold coverage.

The filtered data were used to assemble the genome sequence using both the SMRT analysis pipeline and the Canu assembler. The polished assembly generated using the HGAP2 and HGAP3 workflows of the SMRT pipeline was then compared with the assembly data obtained from Canu to gain a clear picture as to the quality of sequence assembly (supplementary table S1, Supplementary Material online). Out of 795.2 Mb of data, the HGAP3 workflow produced seven contigs, while HGAP2 and Canu produced six contigs. After the circularization of the contigs, the total size of the assembled genome from HGAP3, HGAP2, and Canu was determined to be 5,428,378, 5,413,947, and 5,414,624 bp, respectively.

After assembling the single SMRT cell of the PacBio-generated sequence, the 4.36 million paired-end (PE), preprocessed reads of MiSeq were used to map onto different assemblies. Overall, about 99.32% of MiSeq reads could be aligned with the assembled sequence. However, the best alignment was seen with the Canu assembly, where 97.38% of reads were aligned concordantly and uniquely, whereas only 1.7% of the reads aligned to more than one position. Therefore, the Canu circulated assembly, which gave a very high-quality reference genome without any gaps or ambiguous nucleotides is taken as a final genome assembly of *S. fuliginis* ATCC 27551 (GCA_006363875.1). The complete genome contains six contigs, consisting of two chromosomes and four plasmids. The sizes of the largest contigs named as chromosome I and chromosome II are 3,844,607 and 1,213,992 bp, respectively. Of four plasmids, only three were circularized successfully, the fourth plasmid remained open-ended. These four indigenous plasmids are named pSF1 (224,736 bp), pSF2 (60,848 bp), pSF3 (43,234 bp), and pSF4 (27,207 bp).

After generating the high-quality genome sequence of *S. fuliginis* ATCC 27551 it was compared with the draft genome sequences of two more strains of *S. fuliginis* available in the NCBI database (supplementary table S2, Supplementary Material online). The genome of *S. fuliginis* DSM 18781 (GCA_004152845.1) is available as 21 contigs. Of these 21 contigs, 10 contigs showed similarity with chromosome 1, and 5 contigs aligned with chromosome 2 of *S. fuliginis* ATCC 27551. Only one contig aligned with the largest plasmid, pSF1, and no sequence information from strain DSM 18781 showed any similarity with the other three indigenous plasmids of strain ATCC 27551.

In contrast, the sequence of *S. fuliginis* ATCC 27551 failed to align properly with the sequence of *S. fuliginis* OMI, which is available as 34 scaffolds (GCA_002723855.1). When we blasted the largest contig of OMI with the genome sequence of ATCC 27551 it matched with the chromosome 1, chromosome 2, and one of the indigenous plasmids, pSF1, indicating erroneous assembly of the sequence of *S. fuliginis* OMI. Since there is no raw data, we could not make use the available sequence to perform comparative genomics.

### Genome Annotation

**Fig. 1.**
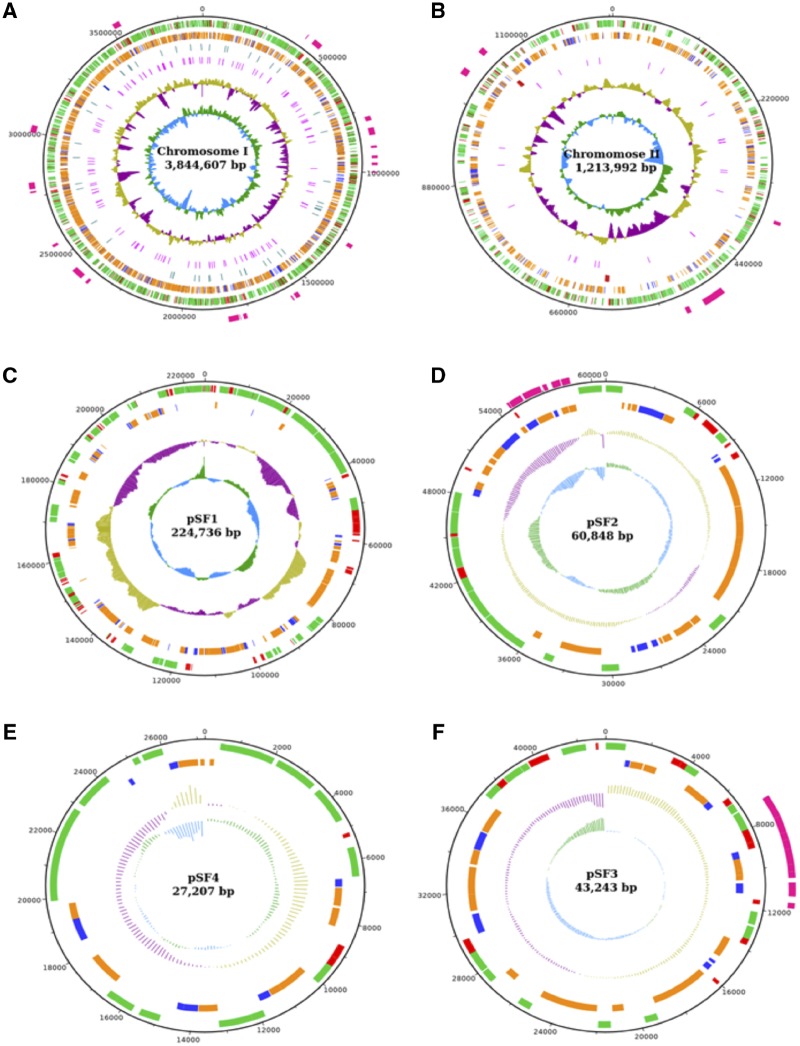
—Circular maps of the chromosomes (A and B) and plasmids (C- F) of *Sphingobium fuliginis* ATCC 27551. The innermost circle depicts the GC-skew of the reverse and forward strands in dodger blue and dark green, respectively. The second innermost circle represents the GC-content of the reverse and forward strands represented in violet and dark yellow, respectively. The third innermost (in pink) represents VNTRs. The fourth circle represents RNA genes (rRNAs in dark blue and tRNAs in light blue). Hypothetical and annotated proteins of the reverse strand are shown in blue and dark orange, respectively, in the fifth circle. The hypothetical (in red) and annotated proteins (in vivid green) of the forward strand are shown in the sixth circle. Island patches have been shown in pink color in the outermost circle. VNTRs are not shown in plasmids. The fourth circle is absent in plasmids(C- F) as the genes coding rRNAs and tRNAs are not present.

The two chromosomes of *S. fuliginis* ATCC 27551 are annotated with 5,164 genes, of which chromosome I contains 4,030 genes and chromosome II 1,134 genes (supplementary table S3, Supplementary Material online). Of the 5,164 genes, 5,100 are protein-coding genes and the remaining 64 genes code for tRNA (55) and rRNA (9) molecules. Chromosome I includes 51 tRNA genes and 3 rRNA genes, whereas chromosome II has only 4 tRNA and 6 rRNA genes. The coding density of the genome is 86.72% and the GC content is 64.37%. The distribution of various features of the sequenced genome, such as functionally assigned genes, hypothetical genes, GC content, GC skew, VNTRs are shown in figures 1*A* and 1*B*.

**Fig. 2.**
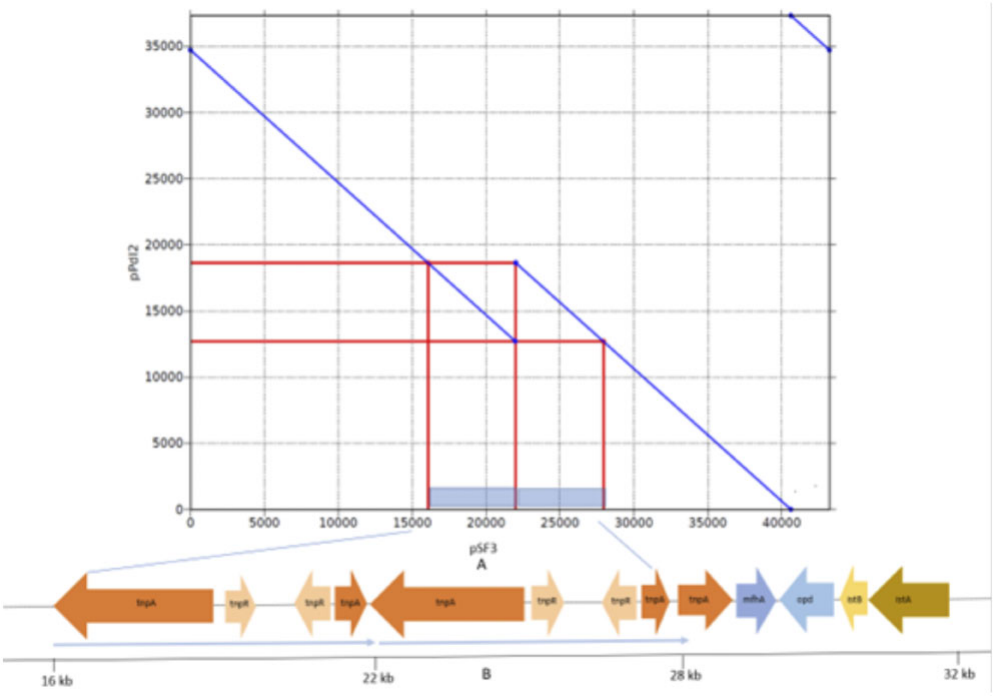
—Graphical representation of the pair-wise alignment of SMRT-generated pSF3 sequence with pPDL2 is shown along with ∼6 kb duplicated region in panel (*A*). The genetic organization of the duplication region is shown in panel (*B*).

Annotation of the four indigenous plasmids pSF1, pSF2, pSF3, and pSF4 identified 393 genes. Among them, plasmid pSF1 contains 239 genes. The other 154 genes are distributed among pSF2 (66), pSF3 (51), and pSF4 (37) (supplementary table S3, Supplementary Material online). All annotated genes code for proteins and no RNA-coding genes were found in any of the four plasmids. The GC content of the plasmids varied from 61.18% to 63.17%, whereas the coding density varied from 81.38% to 84.94% (fig. 1*C*–*F*).

The functional characterization of the proteome of *S. fuliginis* ATCC 27551 was done by performing Clusters of Orthologous Groups (COG) analysis. Out of 5,100 annotated proteins, 2,679 could be grouped into 23 COG categories (supplementary fig. S1, Supplementary Material online). Protein clustered with general cellular functions (R), lipid transport and metabolism (I), energy production and conversion (C), and transcription (K) were enriched in *S. fuliginis* ATCC27551, along with a COG cluster consisting of proteins of unknown function (S). In contrast, just one predicted gene-product was found in the COG cluster related to chromatin structure and dynamics (B) and <50 proteins were allocated to the COG groups W (extracellular structure), D (cell cycle control), N (cell motility), and X (Mobilome).

### Orthology and Phylogenetic Analysis

The orthologous genes were identified in the *Sphingobium* genus by taking 223,547 cumulatively annotated genes found in the genomes of 49 species. Of 15,455 orthologous groups, only 802 were present in all 49 species and hence were designated as a core genome of the *Sphingobium* genus. *Sphingobium* sp. 22B was represented in the highest number of orthologous groups (4,799), whereas the lowest number (2,916) of orthologous groups was found in *Sphingobium* sp. SYK-6. Of 4,786 genes of *S. fuliginis* ATCC 27551, only 4,502 existed as part of known orthologous groups. The remaining 449 genes did not cluster with any of these orthologous groups and hence were considered as singletons. However, six orthologous groups comprising a total number of 13 genes were specific to only *S. fuliginis* ATCC 27551 and were not found in any of the other 49 genomes analyzed. Therefore, these 13 genes and 449 singletons are considered as unique to the genome of *S. fuliginis* ATCC 27551.

A significant proportion of the unique genome of the *Sphingobium* genus codes for transposases and it was found to be the largest orthologous group with 283 genes. The top 15 largest orthologous groups of *Sphingobium* genus are highly enriched and contain a number of paralogous sequences. These orthologous groups code for efflux pumps and membrane transporters.

The phylogenetic inferences of the *Sphingobium* genus were drawn by taking the core genome into consideration. Of 802 orthologous groups, 251 were found to be multicopy orthologs. Thus, only 551 single-copy orthologs were used while constructing the phylogenetic tree for the genus. The phylogenetic tree thus constructed contains three clades. The clade where *S. fuliginis* ATCC 27551 is grouped contains *Sphingobium* sp. kk22, *Sphingobium* sp. YBL2, *Sphingobium* sp. AM, and *Sphingobium* sp. 22B. All of them are involved in degradation of rare and recalcitrant aromatic compounds, such as polyaromatic hydrocarbons (PAHs) and herbicides (Gu et al. 2013; Maeda et al. 2013, 2015; Madueño et al. 2016; Festa et al. 2017). The clustering of *S. fuliginis* ATCC 27551 with PAH-degraders appears to have functional and evolutionary significance. This clade apparently shows considerable distance with hexachlorocyclohexane (HCH)-degrading *Sphingobiumjaponicum* and *Sphingobiumindicum* (supplementary fig. S2, Supplementary Material online).

### Indigenous Plasmids

The plasmids of *S. fuliginis* ATCC 27551 have received considerable attention because they possess the genetic repertoire required for the degradation of OP insecticides and they play a critical role in the horizontal mobility of the *opd* (OP-degradation) gene. *Sphingobiumfuliginis* ATCC 27551 has four plasmids (Mulbry et al. 1986, 1987) and their sizes were previously determined by electrophoretic mobility to be 51 kb (pPDL1), 43 kb (pPDL2), 27 kb (pPDL3), and 23 kb (pPDL4). Among these plasmids, only pPDL2 carries the *opd* gene and plays a role in transferring the *opd* gene to other soil bacteria (Siddavattam et al. 2003, 2019; Pandeeti et al. 2012). Our genome sequence analysis of *S. fuliginis* ATCC 27551 also identified these four plasmids; however, surprisingly, their sizes did not match those determined by electrophoretic mobility (Mulbry et al. 1986, 1987). Based on our sequence analysis, these four plasmids have now been renamed as pSF1 (224,736 bp), pSF2 (60,848 bp), pSF3 (43,234 bp), and pSF4 (27,207 bp) to indicate the source of isolation and to maintain uniformity. Plasmid pSF1 codes for 239 proteins, and of these 89 are hypothetical proteins. All genes required for coding a Type 4 secretory system (T4SS), a typical feature required for mediating horizontal gene transfer (HGT) are found on plasmid pSF1. Interestingly, pSF1 also includes a complete set of genes required for copper uptake and codes for efflux pumps, which are predicted to confer resistance to cobalt–zinc–cadmium. Likewise, plasmid pSF2 also contains genes coding for a functional T4SS. Most of the other predicted genes of plasmid pSF2 code for hypothetical proteins. Interestingly, genes encoding the T4SS are absent on both pSF3 and pSF4. However, these two plasmids have well-conserved *oriT* (origin of transfer) sequences and encode relaxase, which is required for initiating HGT.

The sequence of plasmid pPDL2 was manually determined and coincided with the sequence of pSF3. Both of the sequences showed exact synteny, except in the region spanning 12,680–18,619 bp. This region hits twice to the SMRT assembled sequence of pSF3 indicating the existence of a 5,939 bp duplication in the sequence of pSF3 (fig. 2*A*). The duplication is seen near the *opd* element. In this region, the *opd* and *mfhA* genes coding for an esterase, are flanked by IS*21* and a duplicated Tn*3* (fig. 2*B*). Plasmid pSF3 also contains a functional integration module that can integrate at an attachment (*attB*) site such as those typically found in integrative mobilizable elements. These two unique structural features of plasmid pSF3 strongly suggest it involvement in the lateral transfer of the *opd* gene among soil bacteria.

The genus *Sphingobium* plays a critical role in the mineralization of toxic and recalcitrant organic compounds. Species belonging to this genus have gained prominence due to their bioremediation potential, especially of agricultural soils and dump sites contaminated with HCH. Genome sequences of *S. japonicum*, *Sphingobiumchlorophenolicum*, and *S. indicum* are available in the database (Nagata et al. 2010; Anand et al. 2012; Copley et al. 2012). As we have shown here for *S. fuliginus* ATCC 27551, all of them have two chromosomes. Orthology analysis revealed high pressure on the species to adapt to various environmental conditions. Genes that are under selective pressure are often duplicated and present in high copy-number. Such multicopy genes play a critical role in the adaptability of the species to a particular environment (Wagner 2002). The genes encoding efflux pumps, which confer resistance to heavy metals such as cadmium, zinc, and mercury, are found in multiple copies suggesting the species has the ability to survive in environments polluted with heavy metals (Nies 2003; Ojuederie and Babalola 2017). Another interesting feature of the species is the existence of a high number of outer membrane-associated TonB-dependent receptors (TonRs). TonR are an integral part of TonB-dependent transport (TonBDT) system and are specialized for the transport of nutrients through the energy-deprived outer membrane (Noinaj et al. 2010). Vitamin B12, nickel complexes, and ferric-siderophores are substrates of the TonBDT system. In addition to TonR, the TonBDT system consists a Ton-complex consisting of the proton-motive-force-dependent pump components (ExbB/ExbD) and the energy transducing TonB protein. The Ton complex is conserved in TonBDT. However, the TonR sequences are variable and appear to be substrate-specific (Noinaj et al. 2010). Limited information is currently available on TonBDT making it somewhat difficult to predict to the potential substrate transported (Schauer et al. 2008). However, the number of TonR copies is proportionate to the complexity of the environment (Schauer et al. 2008). Gram-negative bacteria adapted to a pathogenic lifestyle, living in harsh environmental conditions, and those surviving in the mammalian gut contain the highest number of TonRs (Blanvillain et al. 2007; Schauer et al. 2008). These TonRs are implicated to be important in the transport of rare and unique nutrients available in these particular environments. The genome of *S. fuliginis* ATCC 27551 contains 87 copies of the TonR-coding genes, compared with three copies of TonB- and two copies of ExbB/ExbD-coding genes. The existence of such a large number of TonRs with structural diversity indicates the presence of novel TonBDT systems among *Sphingobium* members, perhaps aiding their acquisition of scarce nutrients in soil.

## Supplementary Material

Supplementary data are available at *Genome Biology and Evolution* online.

## Supplementary Material

evz189_Supplementary_Data

## Notes

**Data deposition:** This project has been deposited at NCBI genbank under accession numbers CP041016-CP041021(GCA_006363875.1).

## References

[evz189-B1] Anand S, et al 2012 Genome sequence of *Sphingobium indicum* B90A, a hexachlorocyclohexane-degrading bacterium. J Bacteriol. 194(16):4471–4472. 22843598 10.1128/JB.00901-12 PMC3416241

[evz189-B2] Blanvillain S, et al 2007 Plant carbohydrate scavenging through tonB-dependent receptors: a feature shared by phytopathogenic and aquatic bacteria. PLoS One 2(2):e224. 17311090 10.1371/journal.pone.0000224 PMC1790865

[evz189-B3] Bouchard MF, et al 2011 Prenatal exposure to organophosphate pesticides and IQ in 7-year-old children. Environ Health Perspect. 119(8):1189–1195. 21507776 10.1289/ehp.1003185 PMC3237357

[evz189-B4] Brettin T, et al 2015 RASTtk: a modular and extensible implementation of the RAST algorithm for building custom annotation pipelines and annotating batches of genomes. Sci Rep. 5(1):8365. 25666585 10.1038/srep08365 PMC4322359

[evz189-B5] Copley SD, et al 2012 The whole genome sequence of *Sphingobium chlorophenolicum* L-1: insights into the evolution of the pentachlorophenol degradation pathway. Genome Biol Evol. 4(2):184–198. 22179583 10.1093/gbe/evr137 PMC3318906

[evz189-B6] Festa S, et al 2017 Assigning ecological roles to the populations belonging to a phenanthrene-degrading bacterial consortium using omic approaches. PLoS One 12(9):e0184505. 28886166 10.1371/journal.pone.0184505 PMC5591006

[evz189-B7] Gu T, et al 2013 The novel bacterial N-demethylase PdmAB is responsible for the initial step of N, N-dimethyl-substituted phenylurea herbicide degradation. Appl Environ Microbiol. 79(24):7846–7856. 24123738 10.1128/AEM.02478-13 PMC3837831

[evz189-B8] Guzman GI, et al 2019 Enzyme promiscuity shapes adaptation to novel growth substrates. Mol Syst Biol. 15(4):e8462. 30962359 10.15252/msb.20188462 PMC6452873

[evz189-B9] Hunt M, et al 2015 Circlator: automated circularization of genome assemblies using long sequencing reads. Genome Biol. 16(1):294. 26714481 10.1186/s13059-015-0849-0 PMC4699355

[evz189-B10] Kawahara K, et al 2010 Reclassification of a parathione-degrading *Flavobacterium* sp. ATCC 27551 as *Sphingobium fuliginis*. J Gen Appl Microbiol. 56(3):249–255. 20647682 10.2323/jgam.56.249

[evz189-B11] Kolvenbach BA, et al 2014 Emerging chemicals and the evolution of biodegradation capacities and pathways in bacteria. Curr Opin Biotechnol. 27:8–14. 24863891 10.1016/j.copbio.2013.08.017

[evz189-B12] Kurtz S, et al 2004 Versatile and open software for comparing large genomes. Genome Biol. 5(2):R12. 14759262 10.1186/gb-2004-5-2-r12 PMC395750

[evz189-B13] Langmead B, Salzberg SL. 2012 Fast gapped-read alignment with Bowtie 2. Nat Methods. 9(4):357–359. 22388286 10.1038/nmeth.1923 PMC3322381

[evz189-B14] Li L, et al 2003 OrthoMCL: identification of ortholog groups for eukaryotic genomes. Genome Res. 13(9):2178–2189. 12952885 10.1101/gr.1224503 PMC403725

[evz189-B15] Madueno L, et al 2016 Draft whole-genome sequence of *Sphingobium* sp. 22B, a polycyclic aromatic hydrocarbon-degrading bacterium from Semiarid Patagonia, Argentina. Genome Announc. 4(3): e00488-16. 10.1128/genomeA.00488-16 PMC4891650 27257204

[evz189-B16] Maeda AH, et al 2013 Draft genome sequence of *Sphingobium* sp. strain KK22, a high-molecular-weight polycyclic aromatic hydrocarbon-degrading bacterium isolated from cattle pasture soil. Genome Announc. 1(6):e00911-13. 10.1128/genomeA.00911-13 PMC3820777 24201196

[evz189-B17] Maeda AH, et al 2015 *Sphingobium barthaii* sp. nov., a high molecular weight polycyclic aromatic hydrocarbon-degrading bacterium isolated from cattle pasture soil. Int J Syst Evol Microbiol. 65(9):2919–2924. 26012583 10.1099/ijs.0.000356

[evz189-B18] Mulbry WW, et al 1986 Identification of a plasmid-borne parathion hydrolase gene from *Flavobacterium* sp. by southern hybridization with opd from *Pseudomonas diminuta*. Appl Environ Microbiol. 51(5):926–930. 3015022 10.1128/aem.51.5.926-930.1986 PMC238989

[evz189-B19] Mulbry WW, et al 1987 Physical comparison of parathion hydrolase plasmids from *Pseudomonas diminuta* and *Flavobacterium* sp. Plasmid 18(2):173–177. 2829255 10.1016/0147-619x(87)90046-1

[evz189-B20] Mulbry WW, Karns JS. 1989 Parathion hydrolase specified by the Flavobacterium opd gene: relationship between the gene and protein. J Bacteriol. 171(12):6740–6746. 2556372 10.1128/jb.171.12.6740-6746.1989 PMC210571

[evz189-B21] Nagata Y, et al 2010 Complete genome sequence of the representative gamma-hexachlorocyclohexane-degrading bacterium *Sphingobium japonicum* UT26. J Bacteriol. 192(21):5852–5853. 20817768 10.1128/JB.00961-10 PMC2953701

[evz189-B22] Nies DH. 2003 Efflux-mediated heavy metal resistance in prokaryotes. FEMS Microbiol Rev. 27(2–3):313–339. 12829273 10.1016/S0168-6445(03)00048-2

[evz189-B23] Noinaj N, et al 2010 TonB-dependent transporters: regulation, structure, and function. Annu Rev Microbiol. 64(1):43–60. 20420522 10.1146/annurev.micro.112408.134247 PMC3108441

[evz189-B24] Ojuederie OB, Babalola OO. 2017 Microbial and plant-assisted bioremediation of heavy metal polluted environments: a review. Int J Environ Res Public Health. 14(12): E1504. 10.3390/ijerph14121504 PMC5750922 29207531

[evz189-B25] Pandeeti EV, et al 2012 Multiple mechanisms contribute to lateral transfer of an organophosphate degradation (opd) island in *Sphingobium fuliginis* ATCC 27551. G3 (Bethesda) 2(12):1541–1554. 23275877 10.1534/g3.112.004051 PMC3516476

[evz189-B26] Schauer K, et al 2008 New substrates for TonB-dependent transport: do we only see the ‘tip of the iceberg’? Trends Biochem Sci. 33(7):330–338. 18539464 10.1016/j.tibs.2008.04.012

[evz189-B27] Sethunathan N, Yoshida T. 1973 A *Flavobacterium* sp. that degrades diazinon and parathion. Can J Microbiol. 19(7):873–875. 4727806 10.1139/m73-138

[evz189-B28] Siddavattam D, et al 2003 Transposon-like organization of the plasmid-borne organophosphate degradation (opd) gene cluster found in *Flavobacterium* sp. Appl Environ Microbiol. 69(5):2533. 12732518 10.1128/AEM.69.5.2533-2539.2003 PMC154515

[evz189-B29] Siddavattam D, et al 2019 Lateral transfer of organophosphate degradation (opd) genes among soil bacteria: mode of transfer and contributions to organismal fitness. J Genet. 98(1):23. 30945693

[evz189-B30] Stamatakis A. 2014 RAxML version 8: a tool for phylogenetic analysis and post-analysis of large phylogenies. Bioinformatics 30(9):1312–1313. 24451623 10.1093/bioinformatics/btu033 PMC3998144

[evz189-B31] Wagner A. 2002 Selection and gene duplication: a view from the genome. Genome Biol. 3(5):reviews1012. 12049669 10.1186/gb-2002-3-5-reviews1012 PMC139360

