# Supplementary material for: Genome Organization and Adaptive Potential of Archetypal Organophosphate Degrading Sphingobium fuliginis ATCC 27551
Source: Genome Biol Evol. 2019 Aug 27;11(9):2557–62. doi: 10.1093/gbe/evz189 (PMC6934885; doi:10.1093/gbe/evz189)
Supplement: evz189_Supplementary_Data [file evz189_supplementary_data.docx]

**Supplementary Material.**

**Methodology**

**Whole genome sequencing and assembly**

Whole genome was sequenced initially using the Illumina platform and later the genome was again sequenced using the Pacific Biosciences platform, a third-generation sequencing platform. A single SMRT was used to generate long reads using P6-C4 chemistry on PacBio RS II. PacBio data were assembled using Canu v 1.3 (Koren, et al. 2017) and HGAP v2 and HGAP v3 of the SMRT analysis pipeline V2.303 (Chin, et al. 2013). Assembled contigs from all software packages were circularized by circulator v1.4.0 (Hunt, et al. 2015).

**Assessment of base-call accuracy in the assembly**

The genomic DNA from *S.* *fuliginis* was also sequenced using the Illumina platform (MiSeq). MiSeq data were further pre-processed using trimmotatic and cleaned reads were mapped onto assemblies using bowtie2 (Langmead and Salzberg 2012). Variations between MiSeq data and Canu contigs were annotated using SAMtools and BCFtools (Li, et al. 2009).

**Genome annotation**

RAST server (Brettin, et al. 2015) was used for structural and functional annotation of the genome. Repeats in the genome sequence were identified using trf version 4.09 (Benson, 1999) and islands were deciphered using island viewer v 0.3 (Dhillon, et al. 2015). The KEGG database (Kanehisa, et al. 2017) was searched through blastkoala (Kanehisa, et al. 2016) for functional annotation and pathway association.

**Cluster of Orthologous Group (COG) analysis**

COG profiles were obtained from the conserved domain database (CDD) (Marchler-Bauer, et al. 2010). The whole proteome was subjected to a search against the COG profiles using RPS-BLAST v2.4.1 with an e-value cut-off of 0.01. Protein sequences having at least 25% identity and 70% alignment length with the corresponding COG profile were assigned to the functional group.

**Orthology and Phylogenetic analysis**

All *Sphingobium* genomes available in the public domain were downloaded from the PATRIC database. A proteome data analysis of the 49 species of the *Sphingobium* genus and *S. fulinigis* were used for orthology analysis. Orthologous genes were clustered using Orthomcl v1.4 (Li, et al. 2003) on whole set of species. Orthomcl was run with the strict parameter of “Percent Match Cut-off”, “Percent Identity Cut-off” and “P-value Cut-off”, set at 70%, 40 % and 1 e-10, respectively.

Phylogenetic analyses were performed on the whole set of *Sphingobium* species using single-copy orthologues. Single-copy orthologous groups were identified and single-copy genes were extracted from each species using Perl script developed in-house . Single-copy genes of each orthologous group were independently aligned using MAFFT (Katoh and Standley, 2013). Alignments of each group were concatenated using G-BloCk v.0.9 (Talavera and Castresana, 2007). RAxML (Stamatakis 2014) was used to infer phylogenetic relatedness. The PROTGAMMAWAGF model of substitution, which actually represents the four gamma-distributed heterogeneity rate categories and estimated empirical residue frequency of residue, was used for branch divergence. The phylogenetic tree was visualized using Dendroscope (Huson, et al. 2007).

**Comparison with sequenced Plasmid**

The sequence of one of the *S. fuligins* plasmids, pPDL2, is available in the NCBI database with an accession number JX312671.1 (Pandeeti, et al. 2012). The sequence of pPDL2 was then compared with the in-house-assembled plasmids using Nucmer of Mummer3 package (Kurtz, et al. 2004). Gnuplot (Racine 2006) was used to generate a dotplot to compare the synteny among the plasmids.

**References:**

1. Benson G. 1999. Tandem repeats finder: a program to analyze DNA sequences. *Nucleic Acids Res.* 27(2):573–80. doi: 10.1093/nar/27.2.573
2. Brettin T, Davis JJ, et al. 2015. RASTtk: a modular and extensible implementation of the RAST algorithm for building custom annotation pipelines and annotating batches of genomes. *Sci Rep.* 5:8365. doi: 10.1038/srep08365
3. Chin CS, Alexander DH, et al. 2013. Nonhybrid, finished microbial genome assemblies from long-read SMRT sequencing data. *Nat Methods.* 10(6):563-9. doi:10.1038/nmeth.2474
4. Dhillon BK, Laird MR. 2015. IslandViewer 3: more flexible, interactive genomic island discovery, visualization and analysis. *Nucleic Acids Res.* 43(W1):W104-8. doi: 10.1093/nar/gkv401
5. Hunt M, De Silva N, et al. 2015. Circlator: automated circularization of genome assemblies using long sequencing reads. *Genome Biol.* 16:294. doi: 10.1186/s13059-015-0849-0
6. Huson DH, Richter DC, et al. 2007. Dendroscope: An interactive viewer for large phylogenetic trees. *BMC bioinformatics.* 8:460. doi:10.1186/1471-2105-8-460
7. Jones P, Binns D, et al. 2014. InterProScan 5: genome-scale protein function classification. *Bioinformatics* 30(9):1236-40. doi: 10.1093/bioinformatics/btu031
8. Kanehisa M, Furumichi M, et al. 2017. KEGG: new perspectives on genomes, pathways, diseases and drugs. *Nucleic Acids Res.* 45(D1): D353-61. doi: 10.1093/nar/gkw1092
9. Kanehisa M, Sato Y, et al. 2016. BlastKOALA and GhostKOALA: KEGG Tools for Functional Characterization of Genome and Metagenome Sequences. *J Mol Biol.* 428(4):726-31. doi: 10.1016/j.jmb.2015.11.006
10. Katoh K, Standley DM. 2013. MAFFT multiple sequence alignment software version 7: improvements in performance and usability. *Mol Biol Evol.* 30(4):772-80. doi: 10.1093/molbev/mst010
11. Koren S, Walenz BP, et al. 2017. Canu: scalable and accurate long-read assembly via adaptive k-mer weighting and repeat separation. *Genome Res.* 27(5):722-36. doi: 10.1101/gr.215087.116
12. Kurtz S, Phillippy A, et al. 2004. Versatile and open software for comparing large genomes. *Genome Biol.* 5(2): R12. doi: 10.1186/gb-2004-5-2-r12
13. Langmead B & Salzberg SL. 2012. Fast gapped-read alignment with Bowtie 2. *Nat Methods.* 9(4):357-9. doi: 10.1038/nmeth.1923
14. Li H, Handsaker B, et al. 2009. The sequence alignment/map format and SAMtools. *Bioinformatics*. 25(16):2078-9. doi: 10.1093/bioinformatics/btp352
15. Li L, Stoeckert CJ, et al. 2003. OrthoMCL: identification of ortholog groups for eukaryotic genomes. *Genome Res.* 13(9):2178-2189. doi: 10.1101/gr.1224503
16. Marchler-Bauer A, Lu S, et al. 2010. CDD: a Conserved Domain Database for the functional annotation of proteins. *Nucleic Acids Res.* 39(suppl_1): D225-9. doi: 10.1093/nar/gkq1189
17. Racine J. 2006. gnuplot 4.0: a portable interactive plotting utility. *J. Appl. Econ.* 21(1):133-41. doi: 10.1002/jae.885
18. Stamatakis A. 2014. RAxML version 8: a tool for phylogenetic analysis and post-analysis of large phylogenies. *Bioinformatics.* 30(9):1312-1313. doi: 10.1093/bioinformatics/btu033
19. Talavera G, Castresana J. 2007. Improvement of phylogenies after removing divergent and ambiguously aligned blocks from protein sequence alignments. *Syst Biol*. 56(4):564-577. doi: 10.1080/10635150701472164
